# White matter microstructure and verbal fluency

**DOI:** 10.1007/s00429-022-02579-7

**Published:** 2022-10-17

**Authors:** Natalia Egorova-Brumley, Chen Liang, Mohamed Salah Khlif, Amy Brodtmann

**Affiliations:** 1 Melbourne School of Psychological Sciences, University of Melbourne, Melbourne, Australia; 2 Department of Speech Pathology, University of Melbourne, Melbourne, Australia; 3 Cognitive Health Initiative, Central Clinical School, Monash University, Melbourne, Australia; 4 The Florey Institute of Neuroscience and Mental Health, University of Melbourne, Melbourne, Australia

**Keywords:** Verbal fluency, COWAT FAS, Category fluency animals, Fixel-based analysis (FBA), White matter, Superior longitudinal fasciculus

## Abstract

Poor performance on verbal fluency tasks is associated with an increased risk of post-stroke cognitive impairment. Grey matter regions supporting verbal fluency have been identified via lesion–symptom mapping, but the links between verbal fluency and white matter structure remain less well described. We examined white matter correlates of semantic (Category Fluency Animals) and phonemic or lexical fluency (COWAT FAS) after stroke, accounting for stroke severity measured with the National Institutes of health Stroke Scale (NIHSS), age, sex, and level of education. White matter fibre density and cross-section measures were automatically extracted from 72 tracts, using MRtrix and TractSeg software in 72 ischaemic stroke survivors assessed 3 months after their event. We conducted regression analyses separately for phonemic and semantic fluency for each tract. Worse semantic fluency was associated with lower fibre density in several tracts, including the arcuate fasciculus, superior longitudinal fasciculus, inferior occipito-frontal fasciculus, inferior longitudinal fasciculus, optic radiation, striato-occipital, thalamo-occipital tracts, and inferior cerebellar peduncle. Our stroke sample was heterogenous with largely non-overlapping and predominantly right-lateralised lesions (lesion distribution: left *N* = 27, right *N* = 43, bilateral *N* = 2), dissimilar to previous studies of verbal fluency. Yet, the tracts we identified as correlates of semantic fluency were all left-lateralised. No associations between phonemic fluency performance and fibre density metrics in any of the white matter tracts we extracted survived correction for multiple comparisons, possibly due to the limitations in the selection of tracts and sample characteristics. We conclude that when accounting for the effects of stroke severity, sex, age, and education, semantic fluency is associated with white matter microstructure in the left arcuate fasciculus, superior longitudinal fasciculus, and several occipital tracts, possibly reflecting the disconnection in the sagittal stratum. Our results obtained with fixel-based analysis, complement previous findings obtained with lesions–symptom mapping and neurodegenerative approaches.

## Introduction

Verbal fluency tests are often used to assess language and executive function. Verbal fluency is dependent on information retrieval from our memory, and hence entrains executive cognitive processes, such as self-monitoring, working memory, attentional set shifting, and both selective attention and inhibition. ﻿ Phonemic and semantic fluency are known to be associated both with increased stroke risk (Brady et al. 2001; Levine et al. 2015; Heshmatollah et al. 2020) and cognitive decline after stroke (Babulal 2017; Shaheen et al. 2019).

Prior studies using lesion–symptom mapping have primarily focused on grey matter correlates of verbal fluency and highlighted the importance of frontal and temporal areas (Baldo et al. 2006). They emphasised that phonemic and semantic fluency shows some overlap in the underlying neural correlates, but also recruits distinct regions depending on the task. Specifically, phonemic and semantic fluency was found to both depend on the integrity of the left inferior-frontal gyrus, insula (Biesbroek et al. 2016), and the parietal cortex (Baldo et al. 2006; Chouiter et al. 2016), with additional frontal regions, e.g., middle frontal gyrus and operculum, critical for word retrieval constrained by phonology, and the left ﻿temporal cortex subserving word retrieval constrained by semantics (Baldo et al. 2006; Biesbroek et al. 2016).

Less is known about the role of white matter structure in specifically supporting verbal fluency, although there is substantial evidence of the perisylvian tracts’ role in aphasia and aphasia recovery (Forkel et al. 2014; Forkel and Catani 2018). White matter connections could be relevant for verbal fluency in a variety of ways: (1) for action initiation, such as frontal aslant tract (FAT) and fronto-striatal fibres from the SMA, preSMA, and posterior IFG (Aron et al. 2007; Catani et al. 2013; Rech et al. 2016); (2) for executive and semantic control over conceptual knowledge, such as the IFOF (Duffau et al. 2005; Giampiccolo et al. 2022); (3) for lexical retrieval and conceptual knowledge, such as the ILF, uncinate, and anterior temporal lobe connections (Agosta et al. 2010; Basilakos et al. 2014a; Herbet et al. 2016; Giampiccolo et al. 2022); (4) phonological encoding, such as the arcuate fasciculus (Duffau et al. 2002; Giampiccolo and Duffau 2022); (5) for articulation and auditory–motor transformation, such as the SLF III/anterior segment of the arcuate fasciculus (Duffau et al. 2003; Catani and Bambini 2014; Giampiccolo and Duffau 2022) and the corticobulbar tract (Simonyan et al. 2016).

Approaches based on neurodegenerative diseases, such as understanding the patterns of degeneration in primary progressive aphasia (PPA), have revealed at least two distinct routes to anomia (Mesulam et al. 2015, 2021). One, constituting the leading cause of anomia in semantic PPA, is based on impaired word comprehension and arises in association with anterior and middle temporal atrophy. The other route, associated with impaired retrieval with relatively intact word comprehension as in logopenic PPA, is linked to the temporoparietal junction. In terms of the underlying white matter, the semantic variant PPA has been associated with bilateral alterations in the inferior longitudinal fasciculus and uncinate fasciculus, while the logopenic variant PPA has been linked with mostly left-sided alterations in the inferior longitudinal fasciculus, uncinate fasciculus, superior longitudinal fasciculus, and subcortical projections (Mahoney et al. 2013).

Several previous lesion–symptom mapping studies have identified that phonemic and semantic fluency deficits are associated with damage to anterior white matter tracts including the external capsule, superior and anterior ﻿corona radiata, superior longitudinal fasciculus, inferior fronto-occipital fasciculus, uncinate fasciculus, frontal aslant tract, and anterior thalamic radiations (Chouiter et al. 2016; Li et al. 2017). Thye et al. (Thye et al. 2021) implicated damage to white matter tracts as the primary correlate for fluency deficits, over and above the contribution of frontal or temporal grey matter cortical regions, particularly for semantic fluency. While the authors used improved methods of lesion–symptom mapping, re-examining the shared and distinct neural correlates of semantic and lexical fluency deficits using best practices in reproducibility, they were limited by issues intrinsic to lesion–symptom mapping studies. These include the requirement to obtain a sample with homogenous lesion distribution and sufficient coverage of lesions to make inferences about whole-brain processes, and an associated assumption that there is a direct correspondence between lesion sites and underlying functions. Some evidence suggests that language function is best described as a combination of lesion and network functional connectivity effects (Siegel et al. 2016).

Thye and colleagues (Thye et al. 2021) localised deficits in letter fluency to damage in the anterior corona radiata and superior longitudinal fasciculus. For semantic fluency, they highlighted the anterior corona radiata and smaller clusters in the external capsule and anterior limb of the internal capsule, in addition to portions of the superior longitudinal fasciculus and posterior thalamic radiation. They underscored a substantial overlap between both types of fluency, suggesting that semantic fluency and letter fluency largely rely on the same neural system. However, they did not control for the effects of other variables known to affect verbal fluency, namely, age, sex, level of education (Loonstra et al. 2001), or stroke severity. It, therefore, remains unclear whether the inclusion of these additional factors may have changed what constitutes a white matter correlate of verbal fluency, or could determine whether phonemic and semantic fluency is distinct or overlapping.

Thus, we here aimed to identify white matter networks associated with verbal fluency using non-lesion-based methods to (a) understand whether phonemic and semantic fluency white matter correlates overlap or are completely distinct; and (b) disentangle the contribution of white matter structure and the influence of factors like stroke severity, age, sex, and education on verbal fluency task performance.

## Methods

### Participants

Ischaemic stroke patients from the Stroke Units at Austin Hospital, Box Hill Hospital, and the Royal Melbourne Hospital in Melbourne, Australia, were recruited as part of the Cognition And Neocortical Volume After Stroke (CANVAS) study (Brodtmann et al. 2014). Ethical approval for the CANVAS study was obtained from each hospital’s Human Research Ethics Committee according to the Declaration of Helsinki and all participants provided written informed consent. Additional approval for the current study was obtained from the University of Melbourne’s Psychological Sciences Human Ethics Advisory Group.

All participants in the CANVAS study engaged in a structured interview to obtain information regarding medical history, current medications, and vascular risk factors. Demographic information included age, sex, years of education, and handedness. Participants were excluded if they were unable to give informed consent, had diagnosis of transient ischaemic attack, primary haemorrhagic stroke or venous infarction, or had significant medical comorbidities making it unlikely that they would survive 3 years. Additional exclusion criteria were significant psychiatric history prior to stroke, or MRI ineligibility (e.g., claustrophobia or safety contraindications). At 3 months post-stroke, participants completed MRI scanning and cognitive testing. All participants with pre-processed diffusion-weighted imaging and behavioural data at 3 months post-stroke were included in the current analysis—no additional exclusions were made. Stroke severity was estimated using the National Institutes of Health Stroke Scale (Ortiz and Sacco 2014). Years of education and age were obtained for this study.

### Language assessment

The language assessments included the Token Test task (Spellacy and Spreen 1969), Boston Naming Test (Kaplan et al. 1983), Category Fluency Animals Test, and Controlled Oral Word Association Test (COWAT) with letters F, A, and S (Loonstra et al. 2001) (Table 1). In this study, we examined white matter correlates of phonemic (COWAT FAS) and semantic (Category fluency Animal) fluency only. In both fluency tests, participants were instructed to produce as many exemplars as possible within 1 min. Token Test scores were used to classify the presence of aphasia, with a pre-specified cut-off of < 14 (Spellacy and Spreen 1969).

**Table 1:** Participant demographic information

	Mean (*SD*)	Range
Age (years)	66.71 (12.04)	30–87
Education (years)	12.83 (3.72)	5–24
NIHSS baseline	2.90 (2.33)	0–10
Token Test Score	15.15 (0.96)	10–16
BNT Score	28.05 (1.28)	16–30
Category Fluency Score	19.21 (5.03)	7–31
COWAT_FAS Score	33.18 (12.93)	4–69

	Number of participants
Aphasia based on Token Test (A:N)	14:54*
Gender (M:F)	22:50
Handedness (R:L)	66:6

Note. *NIHSS* National Institutes of Health Stroke Severity, *BNT* Boston naming test, *COWAT* controlled oral word association test, *F* female, *M* male, *R* right, *L* left, *A* aphasic, *N* Non-aphasic, *SD* standard deviation of the mean.*Missing data for 4 participants

### Imaging processing and analysis

All images were acquired on a Siemens 3 T Tim Trio scanner (Erlangen, Germany) with a 12-channel head coil. Sixty diffusion-weighted images (b = 3000 s/mm^2^), and 8 volumes without diffusion weighting (b = 0), were obtained with 2.5 × 2.5 × 2.5 mm^3^ isotropic voxels. A high-resolution anatomical magnetisation prepared rapid acquisition gradient echo (MPRAGE) scan was collected (1 × 1 × 1 mm^3^ voxels) and used to compute intracranial volume using SPM12.

Pre-processing of diffusion-weighted images included denoising, removing Gibbs ringing artefacts, eddy-current distortion and motion correction, bias field correction, and spatial up-sampling. Following these pre-processing steps, white matter fibre orientation distributions (FODs) were computed with Single-Shell 3-Tissue Constrained Spherical Deconvolution (SS3T-CSD), with group averaged response functions for white matter, grey matter, and CSF obtained from the data themselves (Dhollander and Connelly 2016; Dhollander et al. 2019), using MRtrix3Tissue (https://3Tissue.github.io), a fork of MRtrix3 (Tournier et al. 2019). Note that the lesions in stroke participants were not explicitly masked out, but thanks to the SS3T-CSD method, they were automatically characterised as a mixture of WM-like, GM-like, and CSF-like signal. The WM FODs accurately quantify the amount of ‘intact’ WM, while contributions of other (pathological) tissues, such as stroke lesions or white matter hyperintensities, and free water are accommodated in other model compartments (Dhollander et al. 2017). A population template was generated using FOD images from 25 participants. This pre-processing and analysis approach has been used specifically in stroke (Dhollander et al. 2021).

MRtrix produces fibre density (FD) and fibre cross-section (FC) metrics. FD is sensitive to the microstructural (local total intra-axonal volume) and FD to macrostructural (macroscopic fibre-bundle cross-sectional size) changes (Dhollander et al. 2021).

Automated TractSeg tool was used to delineate individual tracts. We extracted 72 tracts of interest in both hemispheres using the default TractSeg pipeline (https://github.com/MIC- DKFZ/TractSeg), limiting the number of streamlines to 10,000. We calculated white matter fibre density and fibre cross-section measures using MRtrix.

**Fig. 1:**
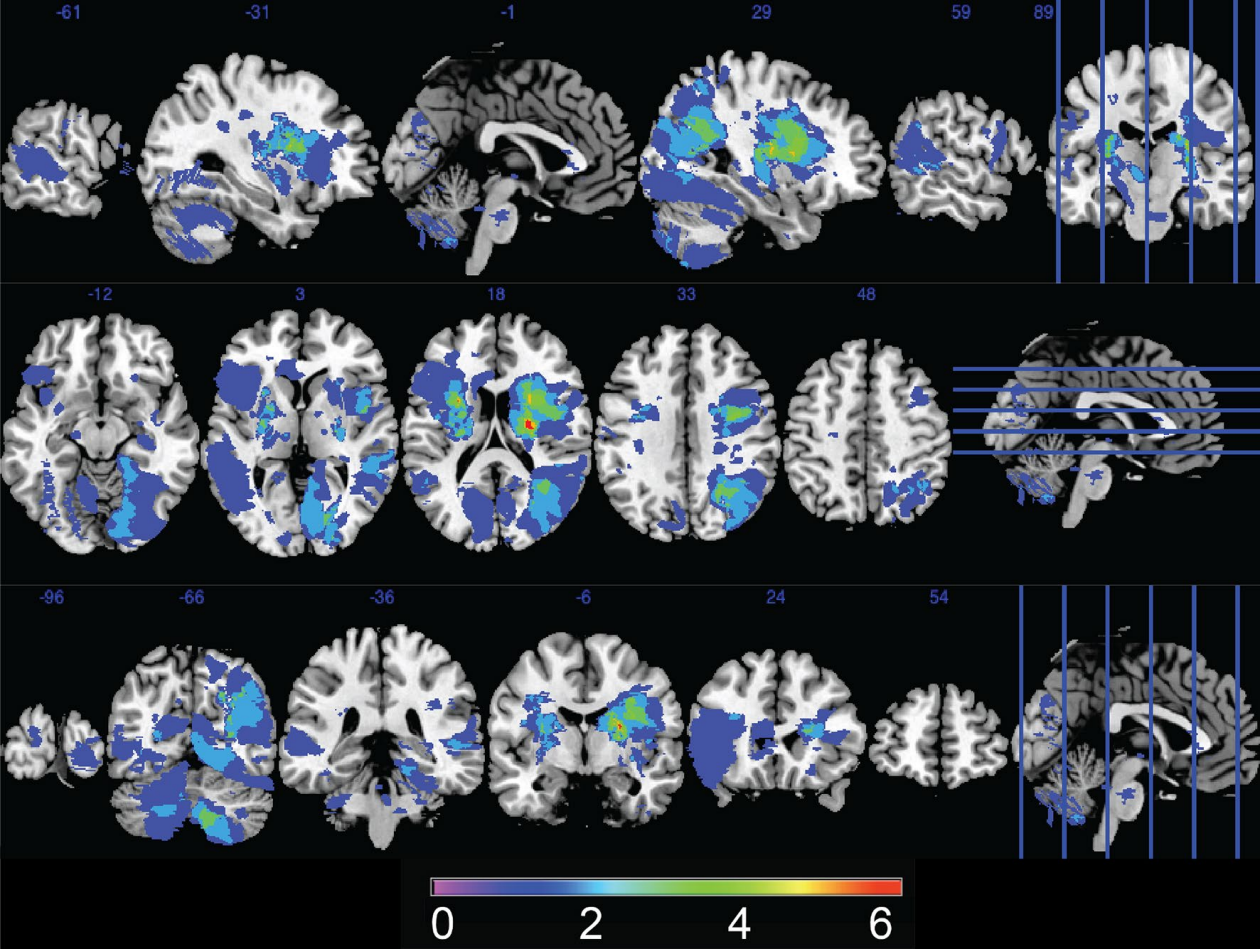
Lesion overlay; the colours show the number of participants with overlapping lesions. Left is on the left

Stroke lesions were manually traced on the high-resolution FLAIR image and checked with clinically acquired acute diffusion-weighted images where available. A stroke neurologist (AB) visually inspected and verified the manually traced images. Binary lesion masks were created and normalised to the MNI template using the Clinical Toolbox SPM extension (Rorden et al. 2012). Lesion maps were prepared using MRIcron software (Rorden et al. 2007); see Fig. 1.

### Statistical analyses

To identify white matter tracts associated with semantic and phonemic fluency, adjusting for stroke severity, age, sex, and level of education, we conducted separate regressions for each of the 72 tracts, using fibre density and fibre cross-section measures. We focused on the association between the fluency scores and white matter metrics, obtaining a *p* value for this association within the model in each tract. Since we repeated this analysis 72 times, we then corrected these *p *values at the false-discovery rate (FDR) level of 0.05, by importing a vector of 72 *p* values into R and using the p.fdr function in the “FDRestimation” package to calculate the FDR-adjusted values.

We additionally explored differences between left- and right-hemisphere stroke subgroups, comparing them on stroke severity, age, sex, and level of education, as well as specifically phonemic and semantic fluency performance. We also repeated white matter analysis for each of the subgroups.

## Results

Seventy-two stroke ischaemic stroke survivors were included in the study. Participants’ demographic characteristics are described in Table 1. Twenty percent of participants (14 of 68) were classified as having aphasia, based on the Token Test scores. Of the 72 participants, *N* = 27 had lesions in the left hemisphere, *N* = 43 in the right hemisphere, and *N* = 2 bilateral. There were no significant differences between the left- and right-hemisphere stroke subgroups in sex, education, NIHSS, and age. Specifically for the fluency tests, semantic fluency was comparable between the groups (Mean(SD) = 18 ± 5 in the left and M = 20 ± 5 in the right-hemisphere group; *p* = 0.16), while phonemic fluency was significantly lower in the left-hemisphere group (left: M = 28 ± 11; right: M = 36 ± 14, *p* = 0.015).

**Fig. 2:**
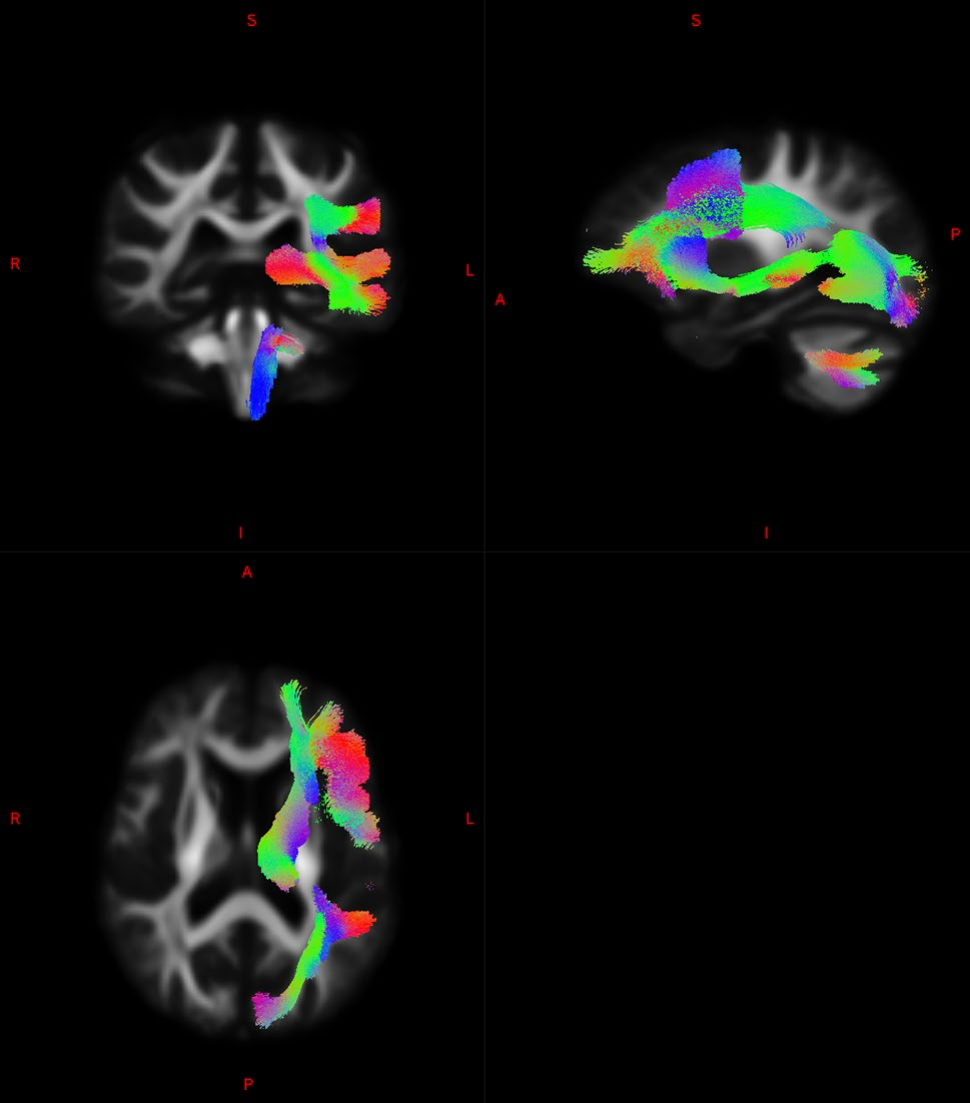
Tracts associated with semantic fluency (FDR-corrected). *R*—right; *L*—left; *A*—anterior; *P*—posterior

Overall, phonemic fluency was not specifically associated with fibre density in any of the tracts at the FDR-corrected level. Semantic fluency was specifically associated with fibre density in 8 left-lateralised tracts surviving the FDR correction, including the arcuate fasciculus (*p* = 0.0036; pFDR = 0.037), inferior cerebellar peduncle (*p* = 0.0047; pFDR = 0.042), inferior occipito-frontal fasciculus (*p* = 0.0032; pFDR = 0.047), inferior longitudinal fasciculus (*p* = 0.0024; pFDR = 0.037), optic radiation (*p* = 0.0033; pFDR = 0.037), superior longitudinal fasciculus III (p = 0.0005; pFDR = 0.035), striato-occipital (*p* = 0.0030; pFDR = 0.037), and thalamo-occipital tracts (*p* = 0.0029; pFDR = 0.037), see Fig. 2. There was a significant (FDR-corrected) effect of education in all tracts. No significant results were observed using the fibre cross-section measure. When the analysis was repeated separately for the left- and right-hemisphere stroke subgroups, no results surviving the FDR correction were observed.

## Discussion

We found that white matter microstructure was not specifically associated with phonemic fluency after stroke. In contrast, while semantic fluency was significantly dependent on the level of education, a number of left-lateralised tracts were specifically associated with this task performance. These findings highlight the important role of white matter health for semantic fluency.

A novel feature of our study was the investigation of the relationship between verbal fluency performance and post-stroke white matter structure using a fixel-based analysis. We derived measures of white matter fibre density and cross-section, compared to more traditionally employed lesion–symptom mapping approaches. Fixel-based analyses ﻿use “fixel” information, which refers to the specific individual fibre population within a voxel. Accounting for fibre orientation distributions, this method likely provides a more sensitive measure of white matter structure, especially in tracts with crossing fibres. Lesion location is often insufficient to explain behavioural deficits, including in language, while network measures appear more useful (Siegel et al. 2016). Our findings based on structural connectivity in a cohort of stroke patients with heterogeneous lesions provide additional insight into the association between white matter health and verbal fluency, complementing previous studies.

Overall, we obtained converging results with prior lesion–symptom mapping studies in white matter and verbal fluency, highlighting the relevance of the superior longitudinal fasciculus III (Fridriksson et al. 2013) and the inferior occipito-frontal fasciculus (Almairac et al. 2015; Chouiter et al. 2016; Li et al. 2017). For semantic fluency, we demonstrated the involvement of the left superior longitudinal fasciculus and the arcuate fasciculus, the anterior segment of which has been shown to be relevant for verbal fluency specifically in patients with post-stroke aphasia (Basilakos et al. 2014a, b). The arcuate fasciculus connects the frontal and temporal areas traditionally referred to as Broca and Wernicke areas (Dick and Tremblay 2012). The superior longitudinal fasciculus III originates from the supramarginal gyrus, ventral to the superior longitudinal fasciculus II and connects to the ventral prefrontal cortex (Thiebaut de Schotten et al. 2011; Parlatini et al. 2017; Howells et al. 2018). These tracts are traditionally associated with language processing (production including articulation, and comprehension). In addition to these tracts, we show the relevance of posterior thalamic tracts only in the semantic task with a different method, by demonstrating thalamo-occipital tract involvement as in the study by Thye and colleagues (Thye et al. 2021).

Other tracts implicated in semantic fluency were observed in the subcortical, occipital, and cerebellar regions, including the inferior fronto-occipital tract, inferior cerebellar peduncle, inferior longitudinal fasciculus, optic radiation, and striato-occipital tracts. The idea that a visuo-spatial mental imagery strategy can be activated during the semantic fluency task (Biesbroek et al. 2016) is consistent with a number of white matter tracts connecting the occipital lobe that were identified in the present study, namely the optic radiation, the inferior-frontal-occipital fasciculus, the inferior longitudinal fasciculus, as well as striato-occipital and thalamo-occipital tracts. The inferior fronto-occipital fasciculus crosses through the temporal lobe and insula, connecting the posterior prefrontal cortex and the posterior temporal region to the occipital lobe for the visual input. The inferior fronto-occipital tract is primarily associated with semantic language processing. In a study of 31 patients with left hemispheric lesions, Almairac and co-authors found an association between lesions in the left inferior fronto-occipital fasciculus and poor semantic, but not phonemic fluency (Almairac et al. 2015). The inferior longitudinal fasciculus primarily connects the occipital lobe of the brain with the anterior temporal lobe, providing critical connections between occipital and anterior temporal areas, both of which are known to contribute to object recognition, and semantic and lexical retrieval processes. It plays an important role in the retrieval of semantic long-term memory and object recognition (Mehta et al. 2016). The inferior cerebellar peduncle is mainly involved in coordination of movement and proprioceptive information and could possibly be also linked with coordinating visuo-spatial imagery.

We also found subcortical involvement in semantic fluency via the striato-occipital and thalamo-occipital tracts. These results further link semantic processing with visual areas and confirm previous studies implicating subcortical regions in verbal fluency (Chouiter et al. 2016). For example, thalamic nuclei have been posited to control the interaction between fronto-opercular and temporo-cortical cortices for the integration of lexico-syntactic with semantic information, passing the resulting signal on to the basal ganglia which are thought to coordinate the release of the language plan into speech (Klostermann et al. 2013). Studies implicating the putamen in fluency tasks (Baldo et al. 2006) are also consistent with our findings of the striato-occipital tract involvement. Furthermore, lower performance on fluency has been previously related to subcortical regions centering on the left thalamus (Biesbroek et al. 2021).

Verbal fluency tasks involve not only purely linguistic but also general cognitive processes, especially executive processes, such as strategic search, attention allocation, ongoing self-monitoring, and inhibition of previously generated responses. An important feature of our study was the inclusion of additional factors known to influence verbal fluency in an attempt to delineate specific linguistic processing. Factors, such as participants’ stroke severity, age, sex, or educational attainment, may reflect semantic memory, executive control, and processing speed contributions to verbal fluency. We found that semantic fluency depends on general intelligence (expressed through years of education) and is consistent with the idea that semantic fluency task engages a search in the semantic or conceptual memory (Baldo et al., 2006). However, even accounting for the effect of education, semantic fluency association with white matter microstructure remained significant.

## Limitations

We have explored semantic and phonemic fluency at 3 months. This is a time point by which post-stroke recovery is relatively stable but still represents an early enough window for rehabilitation interventions. Our cross-sectional study describes white matter tracts associated with verbal fluency at this stage, but it does not address the question whether the trajectory of verbal fluency after stroke changes over time.

One major limitation of our method was that we were only able to extract the 72 tracts available in the automatic TractSeg segmentation. Notably, we could not reliably extract data from the frontal aslant tract that has been previously associated with phonemic fluency (Catani et al. 2013; Basilakos et al. 2014a; Kinoshita et al. 2015). There is also evidence of the role of the subthalamic nucleus (STN) in verbal fluency, e.g., deep brain stimulation of the STN has been shown to improve fluency (Lee et al. 2021). Connections involved in motor initiation, such as preSMA-striatal and IFG-striatal, have also been implicated in fluency (Kinoshita et al. 2015; Rech et al. 2016; Viganò et al. 2022). Specifically, resection of the preSMA in the left hemisphere can cause aphasia together with SMA syndrome (Laplane et al. 1977; Giampiccolo et al. 2021). While we have observed the relevance of thalamo- and striato-occipital tracts in semantic fluency, we could not specifically dissect all of the above tracts known to be relevant for fluency. Furthermore, the use of automatic segmentation also means that several tracts could have been a reflection of the disconnection at the level of a single tract through which they pass, e.g., the sagittal stratum (IFOF, ILF, optic radiation, occipito-striatal, and occipito-thalamic tracts). Therefore, our tract selection could have limited our ability to detect associations between white matter microstructure and phonemic fluency.

Our stroke sample was quite heterogenous, with largely non-overlapping and predominantly right-lateralised lesions, which is unusual for language studies. Unsurprisingly, it was also the left-hemisphere cohort that presented with more impairment in the phonemic fluency task, compared to the right-hemisphere participants. As the number of left-sided strokes in the tested cohort of patients was low, this could have further influenced our ability to identify phonemic fluency associations but not affect our ability to uncover semantic fluency links, since there were fewer participants with stroke lesions in the areas that may contribute to verbal fluency, such as action initiation (i.e., SMA and preSMA) and lexical retrieval (anterior temporal lobe), even though we focused on examining white matter excluding lesions themselves.

Finally, the majority of our stroke survivors had relatively minor stroke and no significant language deficits or clinically diagnosed aphasia, with only 20% of participants possibly having some aphasic features based on their Token Test scores. Thus, our findings may not be generalisable to more severe aphasic populations.

## Conclusions

Our findings of white matter correlates of phonemic and semantic fluency obtained using non-lesion-based structural connectivity methods complement previous studies. We were able to demonstrate that semantic fluency specifically relies on a set of left-lateralised tracts, including the arcuate, superior longitudinal, inferior-frontal and inferior longitudinal fasciculi, as well as striatal and thalamic connections. Our results underscore the critical role of white matter microstructure for subserving semantic fluency.

## Data Availability

The data that support the findings of this study are available on reasonable request from the corresponding author. All requests for raw and analysed data will be reviewed by the CANVAS investigators to determine whether the request is subject to any intellectual property or confidentiality obligations.
